# Characterization of Lipolytic Inhibitor G(0)/G(1) Switch Gene-2 Protein (G0S2) Expression in Male Sprague-Dawley Rat Skeletal Muscle Compared to Relative Content of Adipose Triglyceride Lipase (ATGL) and Comparitive Gene Identification-58 (CGI-58)

**DOI:** 10.1371/journal.pone.0120136

**Published:** 2015-03-26

**Authors:** Patrick C. Turnbull, Sofhia V. Ramos, Rebecca E. K. MacPherson, Brian D. Roy, Sandra J. Peters

**Affiliations:** Department of Kinesiology, Centre for Bone and Muscle Health, Brock University, 500 Glenridge Ave, St Catharines, Ontario, Canada; University of Graz, AUSTRIA

## Abstract

The rate-limiting enzyme in lipolysis, adipose triglyceride lipase (ATGL), is activated by comparative gene identification-58 (CGI-58) and inhibited by the G(0)/G(1) switch gene-2 (G0S2) protein. It is speculated that inhibition of ATGL is through a dose dependent manner of relative G0S2 protein content. There is little work examining G0S2 expression in lipolytic tissues, and the relative expression across oxidative tissues such as skeletal muscle has not yet been described. Three muscles, soleus (SOL), red gastrocnemius (RG), and white gastrocnemius (WG) were excised from 57-day old male Sprague-Dawley rats (n = 9). QRT-PCR was used for mRNA analysis, and western blotting was conducted to determine protein content. ATGL and G0S2 protein content were both greatest in the lipolytic SOL, with the least amount of both ATGL and G0S2 protein content found in the WG. CGI-58 protein content however did not mirror ATGL and G0S2 protein content, since the RG had the greatest CGI-58 protein content when compared to the SOL and WG. When comparing our tissues based on CGI-58-to-ATGL ratio and G0S2-to-ATGL ratio, it was discovered that contrary to oxidative demand, the glycolytic WG had the greatest activator CGI-58-to-ATGL ratio with the oxidative SOL having the least, and no differences in G0S2-to-ATGL across the three muscle types. These data suggest that the content of G0S2 relative to the lipase in skeletal muscle would not predict lipolytic potential.

## Introduction

Adipose triglyceride lipase (ATGL) has been described as the rate-limiting enzyme of lipolysis [1]. ATGL is responsible for catalyzing the removal of the first fatty acid from the glycerol backbone of triglycerides (TG), releasing fatty acids for energy production and subsequently producing diglycerides [1]. It has recently been demonstrated that ATGL has dual regulation, where it is activated by comparative gene identification-58 (CGI-58) and inhibited by the G(0)/G(1) switch gene-2 protein (G0S2) [2–4]. Most studies to date have been conducted in cell culture or in adipose tissue, but relatively little is known about the relationship of these three proteins in other metabolically active tissues, such as skeletal muscle.

It appears that ATGL activity is directly influenced by both CGI-58 and G0S2 since the presence or abundance of these proteins has been demonstrated to alter the hydrolyzing rate of ATGL [2,4,5], with either activation or inhibition, respectively. Yet nothing is known of the ATGL-to-G0S2 or ATGL-to-CGI-58 content ratio amongst non-adipose lipolytic tissues such as skeletal muscle. In G0S2 overexpressing HeLa cells, G0S2 localizes to the lipid droplet membrane and prevents TG hydrolysis in an incremental manner, where increases in relative G0S2 protein content lead to decreases in ATGL catalytic activity [3]. In the same HeLa cell model the loss of G0S2 by knockdown expression caused both basal and stimulated lipolysis to significantly increase [3]. This would suggest that at least in cultured HeLa cells, the content of G0S2 could affect the rate of lipolysis. In skeletal muscle, Parikh et al. [6], demonstrated that G0S2 mRNA expression rose significantly following a hyperinsulinemic euglycemic clamp (*in vivo*), demonstrating the acute responsiveness of the G0S2 gene to elevated insulin concentrations and increased muscle glucose uptake. This would indicate that G0S2 protein content might be expected to decrease lipolysis in response to insulin, although protein content was not measured in this study. Taken together, these data would suggest that the relative content of G0S2 to the ATGL lipase could play an important role in regulating lipolysis. Although the mechanisms behind the regulation of ATGL by G0S2 are unknown, it appears that G0S2 and ATGL are bound together regardless of ATGL activity and lipolysis can be down-regulated even in the presence of CGI-58 [4]. In human adipose tissue following fasting, an increase in adipose ATGL mRNA and protein expression was observed, yet a decrease in G0S2 mRNA and protein expression was also observed [7]. This is understandable since in a fasting state, there is a heightened need for fat mobilization through increased adipose ATGL lipase activity and a decreased need for inhibition of lipolysis. These data suggest that expression of ATGL and G0S2 may play a role in regulating lipolytic rates, at least in adipose tissue. This inverse response in protein content also challenges the notion that G0S2 and ATGL are coordinately regulated, and obviously not bound to a one-to-one stoichiometry in adipose tissue. However, little is known about G0S2 in other tissues (e.g., skeletal muscle) and therefore, further exploration into the contribution of G0S2 inhibition to the regulation of lipolysis is warranted. With tissue-specific differences in metabolic demands, perhaps the relationship between ATGL, CGI-58 and G0S2 can be indicative of known lipolytic profiles across a variety of lipolytic tissues.

However, G0S2 does appear to play an alternate role within mitochondria [8,9]. In cultured ATP depleted hypoxic cardiomyocytes, G0S2 interacts with Complex V (FoF1-ATP synthase) positively regulating ATP production [8]. Interestingly, in human primary fibroblasts placed under TNF-α stress, G0S2 binds to Bcl-2, preventing the Bcl-2/Bax anti-apoptotic heterodimer, thereby promoting apoptosis [9]. While the functions appear to vary, it is interesting to note specific functions of G0S2 within mitochondria as a cell modulator in not only energy production, but also cell viability.

Most studies to date have focused on G0S2 mRNA expression, and few studies have reported G0S2 protein content. Even fewer studies have examined G0S2 in skeletal muscle, so there is little understanding of the basal protein content across varying oxidative muscle tissues [6,10].

The purpose of this study was twofold: 1) to determine ATGL, CGI-58 and G0S2 mRNA expression and protein content in three metabolically different rat skeletal muscles (i.e., soleus, red gastrocnemius and white gastrocnemius); and 2) to determine the G0S2-to-ATGL and CGI-58-to-ATGL ratios within the skeletal muscle tissues to gain information on whether the relative content in these tissues would correspond to the relative reliance on lipolysis and fat oxidation for energy production.

## Methods

### Ethics

All experimental procedures and protocols are approved by the Brock University Animal Care and Utilization Committee and conformed to all Canadian Council on Animal Care guidelines (Animal Utilization Project Protocol permit number: 13-03-03)[11].

### Animals

Male Sprague-Dawley (n = 9; age 57 days, body mass 272 ± 2 g), were used for this study. Rats were ordered from Charles River Laboratories (Quebec, Canada). Animals were housed for 48 hours after delivery in paired cages in a controlled 12:12 hour light to dark cycle in the Brock University Animal Facility. Food was fed ad libitum with access to water at all times.

### Muscle extraction

Rats were anaesthetized by intraperitoneal injections of sodium pentobarbital (6 mg/100 g body wt). Rats were not fasted prior to surgeries; they had access to food up until prior to surgeries. The soleus (SOL), red gastrocnemius (RG), white gastrocnemius (WG) were extracted. The heart was also extracted to act as a positive control for G0S2 [12]. Tissues were immediately snap frozen in liquid nitrogen, and stored at −80°C until analysis.

### Citrate Synthase

As comparison with previous literature, and to ensure the correct muscles were taken during muscle extraction, the SOL, RG, WG and heart were assayed for citrate synthase activity, as previously described by our lab [13]. Briefly, frozen tissue was homogenized in 1 M K_2_HPO_4_ buffer at pH = 8.1. In a cuvette, a real time reaction was initiated by adding triton as well as the substrates oxaloacetate and acetyl CoA into either tissue homogenate or a blank. Subsequently produced free CoASH reacted with 5,5′-dithiobis-(2-nitrobenzoic acid) for colorimetric analysis on a GE Ultrospec 2100 pro spectrophotometer (Baie d’Urfe, Quebec, Canada) at 412 nm [14].

### mRNA extraction

mRNA was extracted using a Trizol method (Invitrogen, Carlsbad, USA). Complementary DNA (cDNA) was reverse transcribed from total RNA using a High Capacity RNA-to-cDNA Kit (Invitrogen, Carlsbad, USA). After reverse transcription, cDNA was kept at −20°C.

### Real Time PCR

SYBR green fluorescent Master Mix was used in all reactions (Invitrogen, Carlsbad, USA) with Acidic Ribosomal Protein 36B4 (36B4) as our endogenous house keeping control gene [15]. Primers were hand selected for ATGL, CGI-58 and G0S2 (Table 1, Integrated DNA Technologies). Real time PCR was carried out using on a Step One Plus thermal cycler (Applied Biosystems). Differences in gene expression between groups were determined using the 2^-ΔΔCT^ method [16].

**Table 1:** Name and sequences of primers.

Primer name	Forward sequence	Reverse sequence
ATGL	5’- AGA CTG TCT GAG CAG GTG GA – 3’	5’ – AGT AGC TGA CGC TGG CAT TC – 3’
CGI-58	5’ – TAC CCG TCA AGG GTC AGT CA – 3’	5’ – CAG CAA GAT CTG GTC GCT CA – 3’
G0S2	5’ – AGC ATG CCT CTT AAG GCT GG – 3’	5’ – GGA TTC GGT GGC ACC TTG AA – 3’
36B4	5’ – ATG TGC AGC TGA TAA AGA CTG GA – 3’	5’ – TGA TCA GCC CGA AGG AGA AG – 3’

### Western Blotting

Sodium dodecyl sulphate polyacrylamide gel electrophoresis was performed on 8% (for CGI-58), 10% (for ATGL) or 15% (for G0S2) running gel, all with 4% stacking gel. Separation was performed at 120V for 90 minutes and transferred onto either a 0.20μm or 0.45μm polyvinylidene difluoride membrane (Amersham Biosciences, Piscataway, NJ) using a 100V current for 1 hour. Blocking was done in either 2% fat free milk in Tris Buffered Saline (TBST) (for CGI-58), 5% fat free milk in TBST (for G0S2), or in 5% bovine serum albumin (BSA) in TBST (for ATGL). Anti-ATGL primary antibody (Cell Signaling Technology, Beverly, Massachusetts, USA #2439s) were diluted at a 1μl: 700μl in 5% BSA in TBST. Anti-CGI-58 primary antibody (Novus Biologicals NB110-41576, Oakville, ON, Canada) was diluted at 1μl: 1000μl in 2% powdered milk in TBST. For G0S2 we used two antibodies, Anti-G0S2 at a dilution of 1μl: 2000μl N-Terminus (Santa Cruz, California, USA sc-133424) and a dilution of 1μl: 2000μl Internal (Santa Cruz, California, USA sc-133423) in 5% powdered milk in TBST. All primary antibody incubations were in 4°C overnight. All secondary antibodies were conjugated with horseradish peroxidase and incubated for 1 hour at either a 1μl: 5000μl (ATGL), 1μl: 10000μl (CGI-58) or 1μl: 20000μl (G0S2) dilution. Exposure was in Immobilon Western Chemiluminescent HRP Substrate (Millipore; Billerica, Massachusetts, USA). Image analysis was performed using band density via ImageJ software produced by the National Institutes of Health (Bethesda, Maryland, USA). A total protein Ponceau stain was used to ensure equal loading (Sigma-Aldrich, Missouri, USA P7170).

### Blocking peptides

Both G0S2 antibodies were pre-incubated overnight at 4°C with specific blocking peptides (N-13: sc-133424 P, Santa Cruz, California, USA. Internal: sc-133423 P, Santa Cruz, California, USA) in concordance with the manufacturers protocol to ensure antibody specificity.

### Statistics

All comparisons were made using a one-way ANOVA with Student-Newman-Keuls post-hoc test using GraphPad Prism version 5 for Windows (GraphPad Software, San Diego California USA). All statistics were performed on our three skeletal muscles in accordance with our *a priori* hypotheses, with reference made to heart tissue within the text and included on the graphs for visual comparison. Significance is reported as p < 0.05.

## Results

### Citrate Synthase

Citrate synthase activity for the heart was roughly 3-fold greater than any of the skeletal muscles. When comparing our SOL, RG and WG samples; our results were consistent with what has been measured previously [13], with RG demonstrating the highest maximal activity (Table 2) (p<0.05).

**Table 2:** Maximal citrate synthase (CS) activity in μmol^.^min^−1.^g^−1^ wet wt.

	SOL	RG	WG	Heart
CS activity	22.3±2.3^a^	34.9±1.9^b^	14.7±1.8^a^	104.0±4.4

Results are mean±SE (n = 8). SOL, soleus; RG, red gastrocnemius; WG, white gastrocnemius. Means designated with the same letter are not significantly different (p<0.05).

### ATGL, CGI-58 and G0S2 mRNA

**Fig 1:**
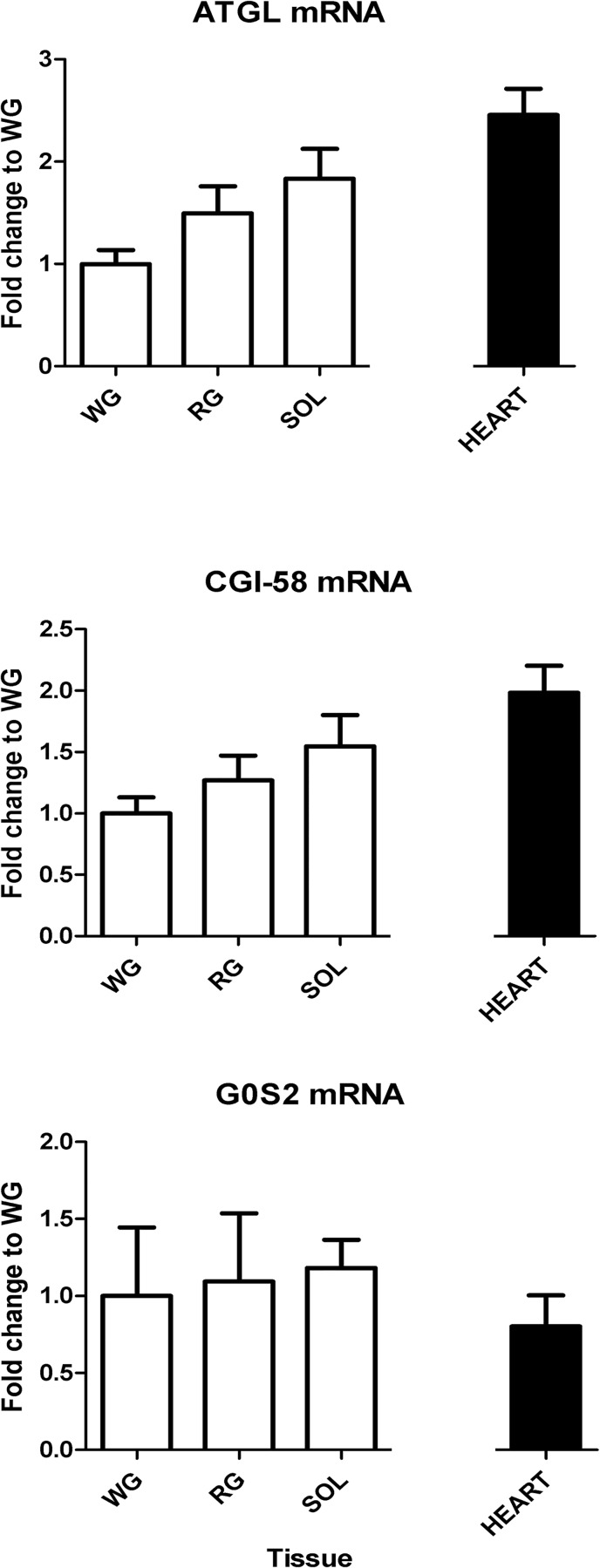
ATGL, CGI-58 and G0S2 mRNA expression in the three skeletal muscles (SOL, RG, and WG and heart, n = 9). Data were calculated according to the delta delta C_T_ method relative to WG as explained in Materials and Methods. Data are reported as mean±SE, and there were no statistically significant differences within the skeletal muscles (p<0.05). SOL, soleus; RG, red gastrocnemius; WG, white gastrocnemius.

Real-time PCR analysis quantified mRNA from SOL, RG, and WG muscles. ATGL, CGI-58 and G0S2 mRNA content was not significantly different across our skeletal muscles (Fig. 1). When assessing heart tissue mRNA, ATGL and CGI-58 mRNA was almost 2-fold greater than the WG.

### ATGL and CGI-58 protein content

**Fig 2:**
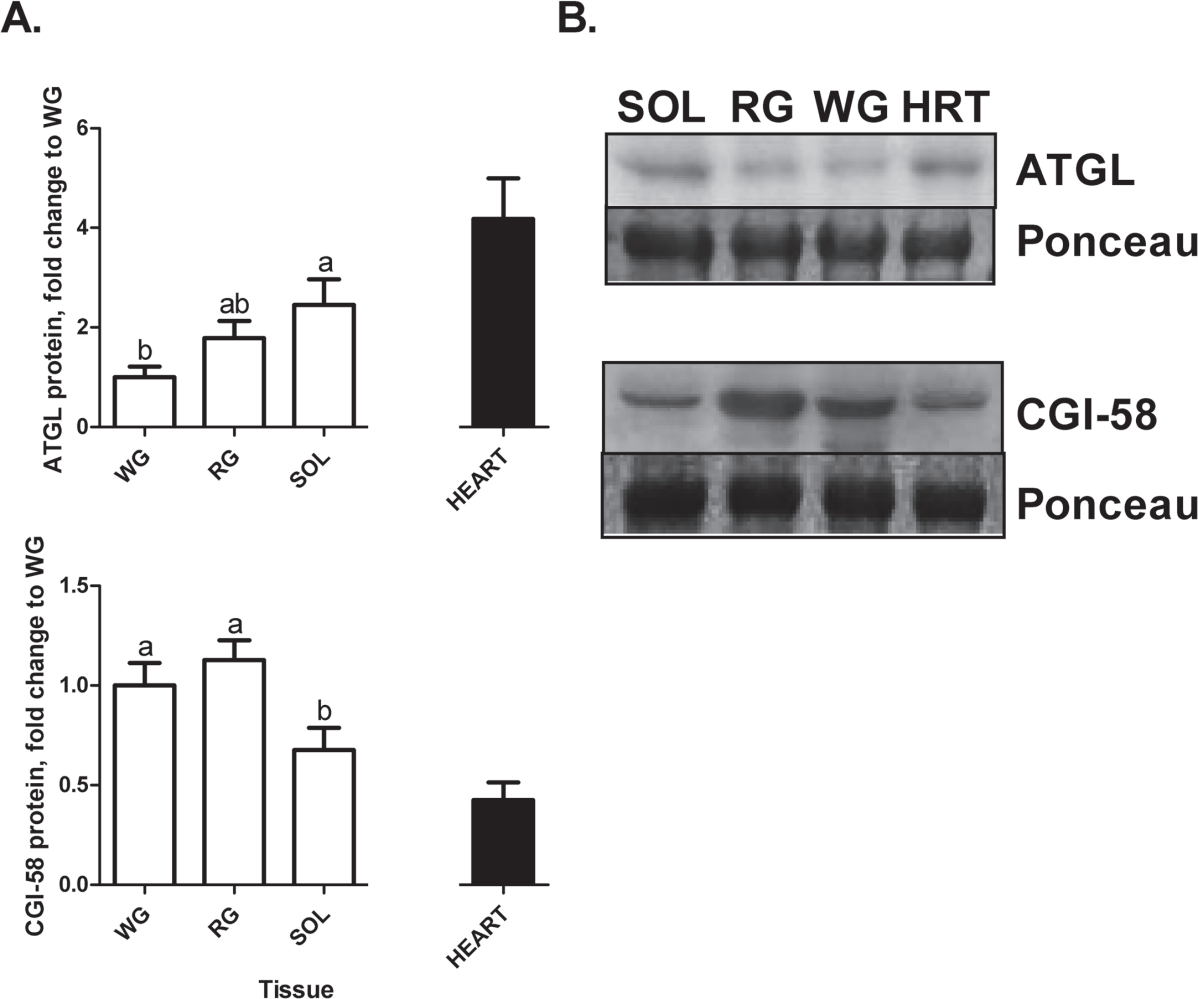
A. ATGL (n = 8) and CGI-58 (n = 7) protein content in the three skeletal muscles (SOL, RG, and WG and heart). Ponceau stain for total protein was used as a loading control. Data are reported as mean±SE and bars with the same letter are not significantly different within the skeletal muscles (p<0.05). Insets: representative blots: lane 1, WG (white gastrocnemius); lane 2, RG (red gastrocnemius); lane 3, SOL (soleus). **B. ATGL and CGI-58 western blot protein bands compared to representative Ponceau bands as equal loading control**.

SOL had approximately 2-fold greater ATGL protein content compared to WG (p<0.05) with RG not being significantly different from either the SOL or the WG (Fig. 2, top panel). The range of activator CGI-58 protein expression (Fig. 2, bottom panel) was markedly different from mRNA expression (Fig. 1, middle panel). RG and WG had the highest CGI-58 protein content, with the SOL having the least. Heart and SOL had similar CGI-58 content.

### G0S2 protein content

**Fig 3:**
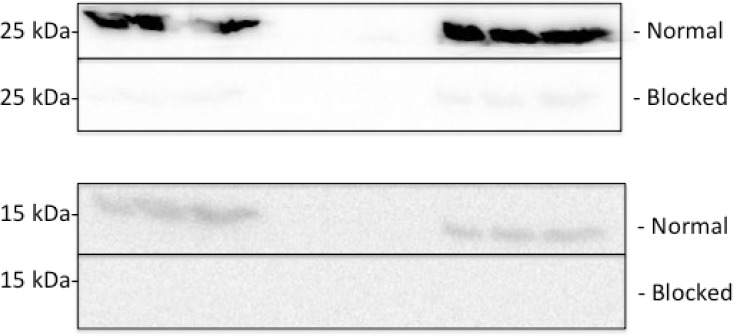
Establishing that G0S2 recognized proteins that migrated at both 15 kDa and 25 kDa. As described in Methods, blots were detected with two antibodies directed against different regions of G0S2 (Santa Cruz, sc-133423, sc-133424) but for the ‘Blocked’ panel, but specific blotting peptides were used (Santa Cruz, sc-133423 P. sc-133424 P). Images were captured at the same time to eliminate bias placed upon exposure times and image editing. Three lanes on the left were mouse SOL (n = 3), three lanes on the right were rat SOL (n = 3). Notice both the 25kDa and the 15kDa band show in both species, and both bands disappear with the antibody pre-incubation with specific blocking peptides.

Although G0S2 has a predicted molecular weight of 11 kDa, we observed two bands that appeared to be G0S2 at ~15 kDa and a very strong band at ~25 kDa. To determine if both the 15 kDa and 25 kDa bands were G0S2 protein we used two antibodies raised against different regions of the protein (Santa Cruz, California, USA, sc-133423, sc-133424), the N-terminus and an internal region, respectively. Both antibodies detected the 25kDa and the 15kDa protein band, but when specific blocking peptides were pre-incubated with the primary antibodies, both bands disappeared or were significantly reduced (Fig. 3). Both the 25kD and the 15kDa band was noticeable in both rat and mouse, with the blocking peptide eliminating both bands in both species, suggesting that the antibodies are specific to both bands.

**Fig 4:**
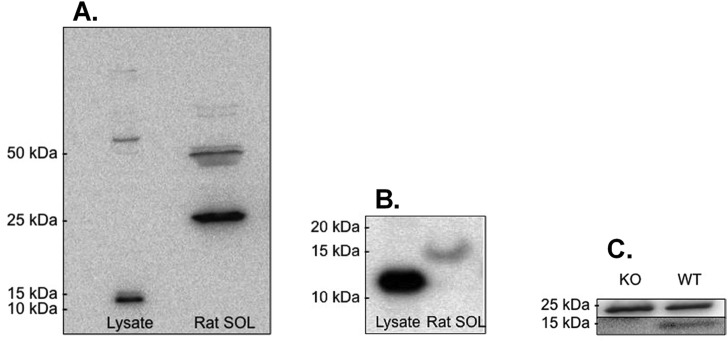
A. G0S2 Overexpressing lysate compared to SOL at 25kDa. Whole blot present shows that the 25kDa band is prominent in SOL with the ~13kDa band present in the lysate and no 25kDa band present. **B. G0S2 Overexpressing lysate compared to SOL at 15kDa**. Notice that the 15kDa band is now visible in SOL lane with the 25kDa cut out prior to exposure. **C. Comparing a wild type to global G0S2 knockout mouse mixed hindlimb muscle for the G0S2 protein (n = 2)**. Notice that in both the WT (wild type) and the KO (knockout) lanes that the 25kDa band is present, yet the 15kDa band is only present in the WT. The top panel represents the 25kDa band, with the bottom panel representing the 15kDa band from the same blot.

To confirm which of the bands was G0S2 we purchased a positive control, a G0S2 293T Cell Transient Overexpression Lysate (H00050486-T01, Abnova, Tapai, Taiwan), and through western blotting demonstrated that G0S2 in the lysate migrated at ~13kDa with no 15kDa band visible when the 25kDa band is present (Fig. 4A). However, when the 25kDa is cut out before incubation with the primary antibody, the 15kDa band becomes visible (Fig. 4B). We then acquired mixed quadriceps skeletal muscle of both wild type (WT) and a global G0S2 knockout (KO) mouse (n = 2), a kind gift from Dr. Jun Liu (Department of Pediatrics and the Kentucky Pediatric Research Institute; University of Kentucky; Lexington, KY USA) [17]. Following western blot analyses, the 25kDa band was present in both WT and KO tissues, but the 15kDa was only present in the WT (Fig. 4C). This confirmed that although the stronger 25 kDa protein was detected by both antibodies, it was not the G0S2 protein. In order to detect the 15kDa band in all tissues, the membrane was cut to eliminate the stronger band at 25 kDa so that the weaker 15 kDa band could be detected.

### 15 kDa G0S2 protein content

**Fig 5:**
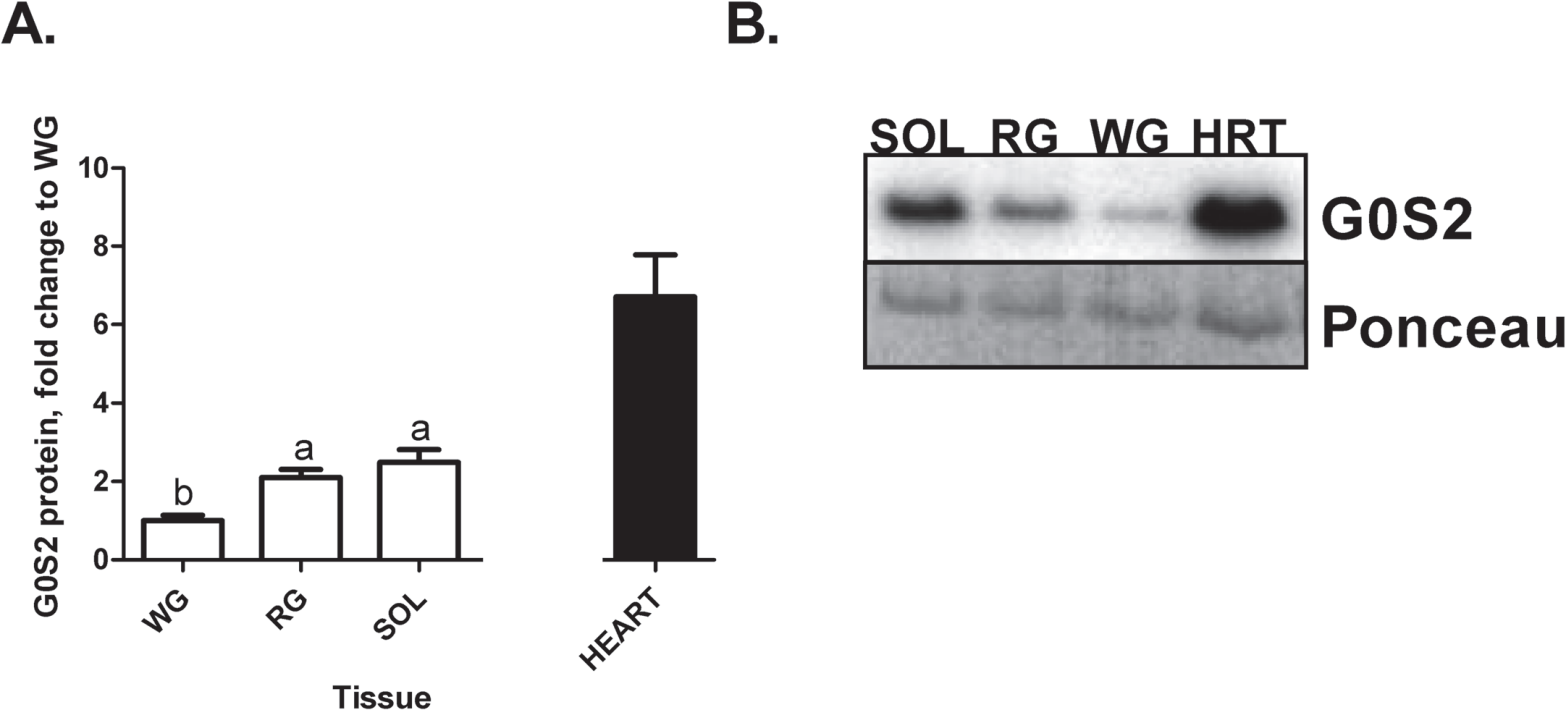
A. G0S2 protein content in the three skeletal muscles (SOL, RG, and WG and heart, n = 8). Ponceau stain was used as a loading control. Data are reported as mean±SE and bars with the same letter are not significantly different within the skeletal muscles (p<0.05). Insets: representative blots: lane 1, WG (white gastrocnemius); lane 2, RG (red gastrocnemius); lane 3, SOL (soleus). **B. 15kDa G0S2 western blot protein bands compared to representative Ponceau bands as equal loading control**.

The G0S2 protein content in SOL and RG was not significantly different, but both had greater than 2-fold G0S2 protein content compared to the WG (p = 0.001; Fig. 5). The heart was roughly 3-fold greater than the SOL and RG.

### Inhibitor G0S2/ATGL and activator CGI-58/ATGL ratios

**Fig 6:**
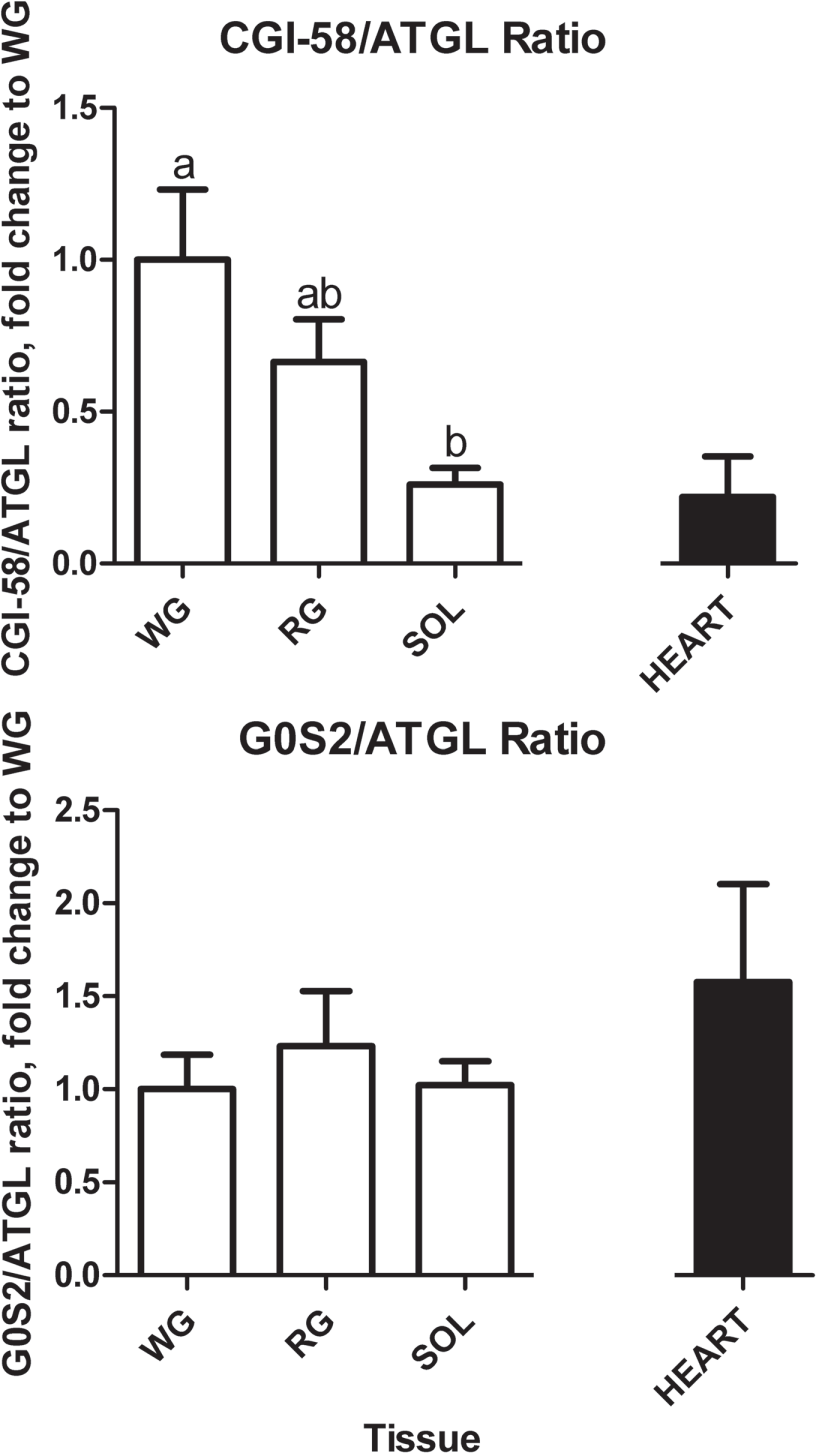
Calculated G0S2-to-ATGL (n = 8) and CGI-58-to-ATGL (n = 7) ratios. Data are reported as mean±SE and bars with the same letter are not significantly different between the skeletal muscles (p<0.05). Please note that ratios were calculated from raw integrations in arbitrary units. Blots were always run in same rat pairs for all the proteins and tissues and every effort to keep exposure times etc., as consistent as possible to keep variation between blots to a minimum. SOL, soleus; RG, red gastrocnemius; WG, white gastrocnemius.

Due to the importance of G0S2 and CGI-58 in regulating ATGL activity, the ratio of the protein content of the inhibitor and activator relative to the lipase content in the three representative muscle fiber types and heart were calculated (Fig. 6). Both WG and RG had an approximately 2-fold greater CGI-58-to-ATGL protein ratio when compared to the SOL (p<0.05, Fig. 6, top panel), however no significant difference in G0S2-to-ATGL protein ratios were observed between the muscle types (Fig. 6, bottom panel). Interestingly, heart tissue had 2-fold greater G0S2-to-ATGL ratio compared to all tissues, yet the lowest CGI-58-to-ATGL.

## Discussion

This study is the first to compare the mRNA expression and protein content of the primary regulatory lipase (ATGL) with its recently identified inhibitor (G0S2) as well as its activator (CGI-58) in non-adipose tissue. To further characterize the expression patterns of these proteins, three metabolically heterogeneous skeletal muscles were chosen to represent the three basic fiber types: SOL (primarily type I, slow-oxidative fibers, 84% type Ia; 7% type IIa and 9% type IIb/x), RG (representative of type IIa fibers, 51% type Ia; 35% type IIa and 14% type IIb/x) and WG (primarily type IIb/x fibers, 0% type Ia; 0% type IIa and 100% type IIb/x) [18]. Several novel findings have been brought forward: 1) we have characterized ATGL, CGI-58 and G0S2 mRNA and protein content across several metabolically different tissues. 2) The ratios of the inhibitor protein (G0S2) and the activator protein (CGI-58) to ATGL in the three fiber types appear to be such that highly oxidative type I SOL had the lowest activator-to-lipase ratio with the WG having the highest. With G0S2 relative to ATGL, the oxidative soleus did not have the lowest content, contrary to our hypothesis, but there was no difference across any tissue, suggesting that relative G0S2 content alone does not regulate ATGL activity.

### ATGL and CGI-58 expression and content in skeletal muscle

ATGL mRNA and protein content followed similar patterns across our array of metabolically different skeletal muscles. As expected, there was the greatest amount of ATGL protein content in SOL, followed by the RG with the least in the WG. This is understandable since it has been previously demonstrated the type I fibers have the greatest capacity for ATGL expression and activity in keeping with their reliance on intramuscular lipolysis [19], and soleus has the highest type I fiber content [18].

The mRNA expression of lipase activator CGI-58 was similar across our array of skeletal muscles. This, however, did not translate to similar protein content. RG had highest CGI-58 protein expression, which corresponds to high oxidative capacity, however the WG has the second highest (although not statistically different from the RG), suggesting that type II muscles have very high CGI-58 regardless of oxidative capacity and reliance on fat as a fuel. In contrast, in 20-week old mice, Badin et al. [20] determined that the heart then the SOL had the highest CGI-58 protein content when compared to a mixed gastrocnemius muscle. The reason for the disparate findings is unclear, but could be suggestive of the protein content of CGI-58 being variable based upon the age and/or species examined. CGI-58relative to ATGL across the three skeletal muscles, we see that the SOL has the lowest activator CGI-58-to-ATGL ratio, with the WG having the highest in spite of the fact that the SOL has the greatest reliance upon intramuscular triglyceride lipolysis as previously demonstrated [21].

### ATGL inhibitor G0S2 mRNA expression and protein content

A novel finding of this study is that G0S2 protein content in sedentary rat skeletal muscle appears to mirror that of ATGL protein content with the SOL and RG having the greatest protein content of both ATGL and G0S2. This is contrary to our hypothesis, since it was postulated from previous work in primarily adipose tissue and cultured cells that the level of the inhibitor G0S2 is indicative of lipolytic reliance or potential of the tissue [3]. Yet our data does not support this since the SOL (which relies heavily on intramuscular triglyceride energy metabolism) has the greatest G0S2 protein content compared to the glycolytic WG. Recently, it has been observed that there was no change in G0S2 protein content in mouse heart tissue following endurance training-induced cardiac hypertrophy [12]. However, this physiological adaptation did not change heart lipid content either, suggesting that the heart remained unchanged in its reliance upon lipid content in response to endurance training [12]. However, these investigators saw that following aortic banding, inducing a model of pathological hypertrophy, there was an increase in intracellular triglycerides and diglyceride storage within the heart. This was accompanied by an increase of heart G0S2 protein content, potentially indicative of decreased rates of lipolysis. However, our interest was in skeletal muscle rather than heart muscle, which in general relies more heavily on intramuscular fuels for energy production.

Our results suggest that, contrary to our hypothesis, muscles with a higher type I fiber content and therefore higher reliance on IMTGs have higher ATGL inhibitor content than muscles with a greater type II content, and the primarily type IIa RG had more potential for lipolytic inhibition than the glycolytic type IIb WG. Further, when expressed relative to ATGL content, type I fibers (SOL) had the same relative amount of G0S2 as both type II muscles (RG and WG). This seemed surprising as SOL relies very heavily on intramuscular triglyceride lipolysis and fat oxidation for energy production during exercise [22,23]. This was demonstrated during exhaustive swimming in male Sprague-Dawley rats where the SOL was more reliant on intramuscular triglycerides compared to both the RG and the plantaris [21–23]. Given this, it would appear that the fiber type that is most dependent on lipolysis has the highest level of potential inhibitory control as well. Therefore, it is not clear why SOL, with its high reliance on intramuscular lipolysis, contains the highest protein content of the inhibitory G0S2. However, in pulse-chase experiments, Dyck et al. [24] indicated that in response to endurance training, rat SOL increased intramuscular triglyceride synthesis, and reduced lipolysis, which could argue for a higher level of control that could be mediated by G0S2 [24,25]. Alternatively, if G0S2 has a more important role in regulating mitochondrial function through either positive regulation of oxidative phosphorylation [8] or through pro-apoptotic pathways [9] our data is consistent with this, given that the more oxidative SOL and RG had the highest G0S2 content.

In summary, it would appear that G0S2 is expressed differently in tissues of varying oxidative capacity and reliance on intracellular lipolysis. However, our results do not support the notion that protein content of the inhibitor G0S2 relative to ATGL content do predict lipolytic potential in skeletal muscle. Therefore, it is clear that regulation of lipolysis is not simply mediated by total content of the important proteins, but through more complicated intracellular or post-translational mechanisms.

## Supporting Information

S1 Table Citrate Synthase raw data. (XLSX)

S2 Table ATGL mRNA and protein raw data. (XLSX)

S3 Table CGI-58 mRNA and protein raw data. (XLSX)

S4 Table G0S2 mRNA and protein raw data. (XLSX)

S5 Table ATGL, CGI-58 and G0S2 protein ratio calculations. (XLSX)
